# Association of C-reactive protein/albumin ratio with mortality in patients with Traumatic Brain Injury: A systematic review and meta-analysis

**DOI:** 10.1016/j.heliyon.2024.e33460

**Published:** 2024-06-22

**Authors:** Yuyang Liu, Yaheng Tan, Jun Wan, Qiwen Chen, Yuxin Zheng, Wenhao Xu, Peng Wang, Weelic Chong, Xueying Yu, Yu Zhang

**Affiliations:** a Center for Evidence-based Medicine, Affiliated Hospital of Chengdu University, Chengdu, Sichuan, China; b Department of Critical Care Medicine, Affiliated Hospital of Chengdu University, Chengdu, Sichuan, China; c West China Hospital, Sichuan University, Chengdu, Sichuan, China; d Public Health Clinical Center of Chengdu, Chengdu, Sichuan, China; e Department of Medical Oncology, Thomas Jefferson University, Philadelphia, PA, USA

**Keywords:** C-Reactive protein/albumin, Ratio, Traumatic brain injury, Mortality, Meta-analysis

## Abstract

**Objective:**

This study examines the C-reactive protein (CRP)/albumin ratio (CAR) as an inflammation-based prognostic score for predicting mortality in patients with Traumatic Brain Injury (TBI).

**Methods:**

We systematically searched the electronic databases PubMed, Embase, and Cochrane up to February 2024. Our inclusion criteria encompassed studies investigating CAR-predicted mortality in patients with TBI. We calculated the Odds Ratio (OR) and associated 95 % confidence intervals (95 % CI) using a random-effects model. Quality assessment of the included studies was appraised using a Newcastle–Ottawa scale.

**Results:**

A total of five studies comprising 1040 patients were included in this meta-analysis. The pooled results indicated that CAR was associated with mortality in patients with TBI (OR = 1.88, 95 % CI: 1.05–3.36, P < 0.0001). The findings of subgroup analysis indicated that the relationship between CAR and mortality in patients with TBI did not vary with the severity of the condition.

**Conclusions:**

CAR emerges as a valuable prognostic tool for mortality in patients with TBI, underscoring its potential role in early risk stratification and management strategies.

## 1 Introduction

The yearly incidence of TBI is estimated at 50 million cases worldwide; thus, approximately half of the global population will have an episode of TBI in their life [1]. TBI is a leading cause of injuries and disabilities and represents a major health concern among young people worldwide [2]. The rates of morbidity and mortality associated with TBI are even higher in low-income and middle-income countries. TBI costs the global economy approximately 400 billion US dollars, representing 0.5 % of the gross world product [1], and it has caused a serious burden on society. Predicting the outcome of patients with TBI can help physicians and patients make appropriate treatment decisions [3].

Following TBI, nerve inflammation can occur, potentially leading to fatal outcomes [4,5]. In clinical practice, it is common to observe elevated C-reactive protein (CRP) levels in critically ill patients, accompanied by hypoalbuminemia [6], indicating acute inflammatory responses and nutritional compromise, respectively. The combination of serum CRP and albumin levels, known as the CAR, serves as a novel, inflammation-based prognostic score [7]. This metric effectively reflects the extent of tissue damage and edema, demonstrating its utility in forecasting the outcomes of various diseases [[8], [9], [10], [11]].

The application of CAR as a biomarker in TBI research has begun to gain traction [12]. Although the significance of CAR in severe diseases has been extensively documented [[13], [14], [15], [16], [17]], its predictive value for mortality among TBI patients remains controversial and understudied. Some studies [[18], [19], [20], [21]] found that elevated CAR levels are correlated with mortality in patients with TBI. However, some studies [22] failed to find correlation. This study seeks to explore the potential of CAR as a predictive marker for mortality in patients with TBI.

## 2 Methods

### 2.1 Search strategy

The PubMed, Embase and Cochrane Library databases, were searched from inception to February 1, 2024 for articles on CAR and outcomes of TBI. Two reviewers (Y.Z and Q.C.) independently assessed the eligibility of the studies. The detailed search strategy used in PubMed was as follows: ‘CRP/Alb ratio’ [All Fields] OR ‘C-reactive protein/albumin ratio’ [All Fields] OR ‘CAR’ [All Fields] OR ‘C-reactive protein’ OR ‘albumin OT CAR OR CRP/Alb’ OR ‘C-reactive protein to albumin ratio, C-reactive protein/albumin ratio’ AND ‘prognosis OR prognostic OR survival OR outcome OR mortality’ AND ‘Brain Injuries, Traumatic’ [Mesh] OR Brain Injury, Traumatic’ [Title/Abstract] OR ‘Traumatic Brain Injuries’ [Title/Abstract]). The references within the retrieved articles were further screened to identify additional, potentially relevant studies.

### 2.2 Inclusion and exclusion criteria

Studies were considered eligible and included if they met all the following criteria: (i) observational studies that documented patients diagnosed with TBI; (ii) research articulating the association between the CAR and mortality among patients suffering from TBI; (iii) provision of adequate data to enable the extraction of OR along with their 95 % CI; (iv) studies published in either English or Chinese.

The exclusion criteria were as follows: (i) studies not involving patients with TBI; (ii) absence of survival outcomes or lack of extractable OR with 95 % CI; (iii) unavailability of the full text, inclusion of meta-analyses, reviews, letters, or duplicate studies; (iv) studies focusing on non-human subjects.

### 2.3 Date extraction

Two investigators (Y.L. and Y.T.) retrieved studies independently, and any discrepancies were solved by discussing with a third reviewer. The extracted relevant data includes the first author, publication year, country, study type, sample size, age, gender, mortality rate, Glasgow coma scale (GCS) score, and Newcastle-Ottawa Scale (NOS) score. The primary outcome studied in the present systematic review and meta-analysis is the association between CAR and mortality in patients with TBI. All authors utilized an electronic data collection form to acquire the necessary information from each article.

### 2.4 Quality assessment

The NOS was adopted to assess the risk of bias in each study included. The assessment was divided into three categories: low risk (7–9), moderate risk (4–6), and high risk (0–3).

### 2.5 Statistical analysis

For statistical analysis, Review Manager 5.3 software was utilized. The OR and their 95 % CI were directly extracted from each study. To assess the heterogeneity among studies, the Cochrane *Q*-test and *I*^2^ statistics were applied. These measures quantify the percentage of total variation across studies that is due to heterogeneity rather than chance, with values exceeding 60 % denoting substantial heterogeneity. To synthesize the OR across studies, a random-effects model was implemented in this meta-analysis. Significance testing was performed using two-tailed P values, with a threshold of *P* < 0.05 considered statistically significant. A forest plot was created to visually represent the degree of heterogeneity among the studies and to provide an estimate of the overall effect size.

## 3 Result

### 3.1 Study selection and characteristics

**Fig. 1:**
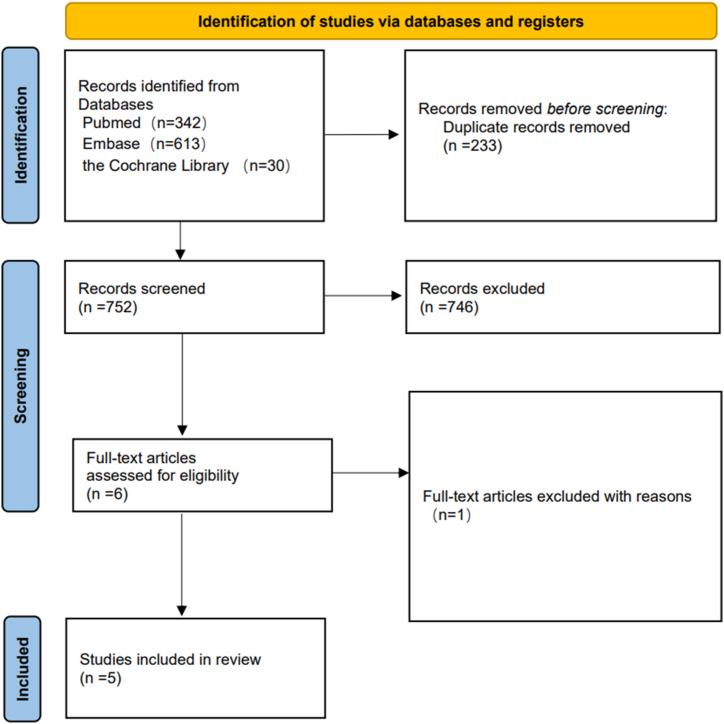
Preferred reporting items for systematic reviews and meta-analyses flow diagram to search and identify included studies.

A total of 1015 articles were identified based on an online database search and manual search, of which 233 duplicated records were removed. By reviewing the titles and abstracts of the articles, 746 articles were removed. After assessing full-text articles, 1 article [23] was excluded owing to a lack of data to measure survival. Finally, 5 eligible articles [[18], [19], [20], [21], [22]] were included in the meta-analysis. A flow diagram is shown in Fig. 1.

Five studies involving 1040 patients were included in this meta-analysis. All studies were published in 2020–2023. Two studies were conducted in Turkey, two in China, and one in South Korea. The sample sizes ranged from 82 to 387 in these studies. All these included studies were of good quality with the NOS scores ≥7. The characteristics of included studies are displayed in Table 1.

**Table 1:** Characteristics of the studies included in the meta-analysis.

Study	Year	Country	Study design	Sample	Age	Sex (male/female)	Mortality (%)	GCS Sore	NOS Score
Wang	2023	China	retrospective observational study	163	55 (47–64)	120/43	34 (20.9)	8 (6–9)	7
Wang	2020	China	retrospective observational study	151	44 (28–56)	119/32	54 (35.8)	6 (4–7)	8
Ozeren	2022	Turkey	retrospective observational study	257	43 (19–91)	221/36	84 (32.7)	NR	7
Gürsoy	2022	Turkey	retrospective observational study	82	49.0 ± 22.69	56/26	23 (28.0)	6.5 ± 4.2	8
June	2022	Korea	retrospective observational study	387	18-64 (16) >65 (223)	265/122	89 (23.0)	NR	8

GCS score: Glasgow Coma Scale score; NOS score; Newcastle-Ottawa Scale score.

**Table 2:** Newcastle–Ottawa scale Score.

Study	Selection				Comparability	Outcome		
	Representativeness of the exposed cohort	Selection of the non exposed cohort	Ascertainment of exposure	Demonstration that outcome of interest was not present at start of study	Comparability of cohorts on the basis of the design or analysis	Assessment of outcome	Was follow-up long enough for outcomes to occur	Adequacy of follow up of cohorts
Wang 2023	*	*	*	*	*	*	*	
Wang 2020	*	*	*	*	*	*	*	*
Ozeren 2020	*	*	*	*	*	*	*	
Gürsoy 2022	*	*	*	*	*	*	*	*
June 2022	*	*	*	*	*	*	*	*

### 3.2 Quality assessment

The quality of the eligible articles was evaluated by NOS (see Table 2). As shown in additional Table 2, the NOS scores of the included studies ranged from 7 to 8 and were regarded as high quality.

### 3.3 CAR and TBI mortality

**Fig. 2:**
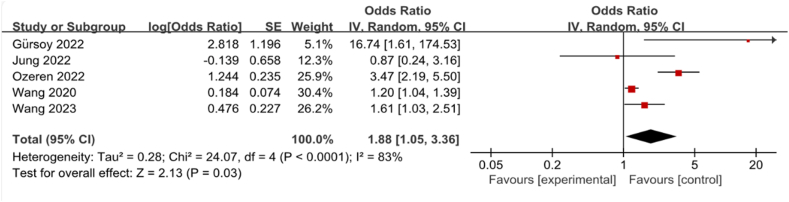
Forest plot of the association between C-Reactive Protein/Albumin Ratio and mortality in patients with Traumatic Brain Injury.

Five studies encompassing a collective total of 1040 patients were examined to explore the association between the CAR and mortality in individuals suffering from TBI. The aggregated findings, depicted in Fig. 2, indicate a significant association between elevated CAR levels and increased mortality rates among patients with TBI (OR = 1.88, 95%CI: 1.05–3.36, *P* < 0.0001). Given the presence of considerable heterogeneity across the studies (*I*^2^ = 83 %), a random-effects model was employed for the analysis.

### 3.4 Subgroup analysis

**Fig. 3:**
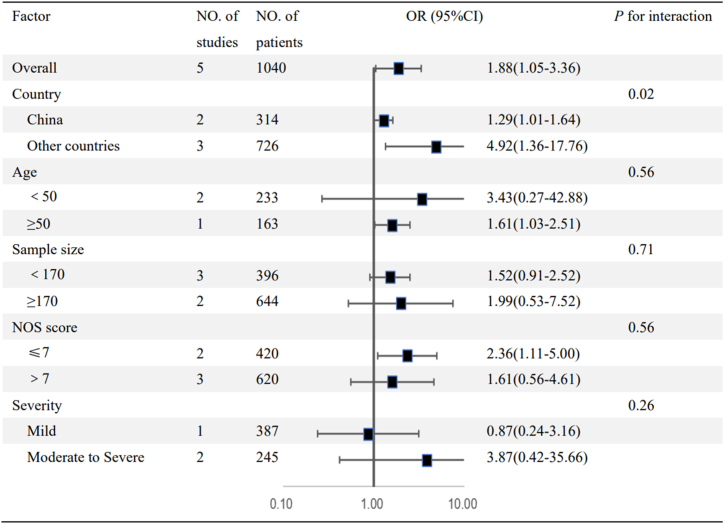
Subgroup analysis of the association between C-Reactive Protein/Albumin Ratio and Traumatic Brain Injury. Cl confidence interval, P: P for interaction for subgroup difference.

Substantial heterogeneity was identified across all included studies (*I*^2^ = 83 %, *P* < 0.05). This heterogeneity was mitigated following subgroup analysis. Subgroup analyses were conducted to determine potential sources of heterogeneity, stratifying by country of included studies, age, sample size, Newcastle–Ottawa scale Score, and severity of patients with Traumatic Brain Injury. The results were summarized in Fig. 3.

### 3.5 Sensitivity analysis

Sensitivity analysis demonstrated that removing any single study did not significantly alter the aggregated outcomes, as shown in Table 3. This indicates the robustness of the pooled results against the potential bias of individual studies.

**Table 3:** Sensitivity analysis of meta-analysis.

	NO. Patients (trials)	OR	95%CI
**All trials**	1040 (5)	1.35	1.18,1.54
**Using a fixed-effect model**	1040 (5)	1.88	1.05,3.36
**Excluding trials with Wang 2023**	877 (4)	1.33	1.16,1.52
**Excluding trials with Wang 2020**	889 (4)	2.28	1.68,3.10
**Excluding trials with Ozeren 2022**	783 (4)	1.24	1.08,1.42
**Excluding trials with Gürsoy 2022**	958 (4)	1.34	1.17,1.53
**Excluding trials with June 2022**	653 (4)	1.36	1.19,1.55

## 4 Discussion

In this study, we scrutinized five studies that aligned with our inclusion criteria [[18], [19], [20], [21], [22]]. Our results show that CAR was a significant predictor of mortality in TBI.

To our knowledge, this is the first meta-analysis to evaluate CAR for predicting mortality in patients with TBI. CAR have been shown to predict a wide range of severe diseases [24]. Notably, Huang [15] et al. have revealed that CAR can accurately predict short-term clinical outcomes in patients with stroke-associated pneumonia (SAP) and acute ischemic stroke (AIS). Akpınar CK [14] et al. have further confirmed the utility of CAR as a prognostic marker for mortality among acute ischemic stroke patients who undergo mechanical thrombectomy. In a related finding, Liu [16] et al. discovered that elevated CAR levels are linked to higher mortality rates and poorer prognoses in sepsis patients. Additionally, Sonsöz MR [17] et al. have demonstrated an association between CAR and increased all-cause mortality in patients hospitalized with acute decompensated heart failure. These studies suggest that underscore the broad applicability of CAR as a predictive tool across a spectrum of critical conditions. However, several studies [[18], [19], [20], [21], [22]] have investigated the potential relationship between CAR and mortality rates in TBI patients, resulting in inconsistent findings. While some research [[18], [19], [20], [21]] indicates a positive association, others report [22] no significant correlation, leading to a lack of consensus in the field.

Our study is not without limitations. Firstly, our analysis was limited to only five eligible studies [[18], [19], [20], [21], [22]], encompassing a total of 1040 patients. This limitation is especially significant for subgroup analyses, where the small sample size may not be adequate to ensure reliable outcomes or to perform in-depth analyses. Additionally, we noted a moderate to high level of heterogeneity among the included studies. This heterogeneity could stem from differences in study methodologies, the severity of patient conditions, and the diverse nationalities of the patient cohorts, potentially leading to variations in the aggregated results.

The results indicate the potential of CAR in predicting the clinical outcomes of TBI, providing valuable insights into patient mortality. To definitively ascertain the predictive accuracy of CAR for mortality in patients with TBI, larger-scale studies are imperative. Future prospective studies should aim to address the limitations of this preliminary research, ultimately refining the clinical applicability of CAR.

## 5 Conclusion

Our meta-analysis highlights the crucial role of CAR levels in predicting mortality in patients with TBI, emphasizing its potential as an early prognostic indicator.

## Data availability declaration

The data related to this study has not yet been stored in a public repository. Data is included in the original articles/supp. material/referenced in article.

## Ethics declarations

The ethical statement is not applicable in this study as this is a systematic review and meta-analysis – this is a secondary analysis of published information.

## Funding

This work is supported by 10.13039/100014717National Natural Science Foundation of China (82271364), the innovation team project of Affiliated Hospital of Clinical Medicine College of Chengdu University (CDFYCX202203)（Yu Zhang)，and the project of Sichuan Science and Technology Bureau (22ZDYF0798), the 1·3·5 project for disciplines of excellence-Clinical Research Incubation Project, 10.13039/501100013365West China Hospital, Sichuan University (21HXFH046), the project of 10.13039/501100020207health commission of Sichuan province (2019HR50), Experimental Teaching Research and Reform Project of Chengdu University (cdsyjg2022022) , and Science and Technology Research Project of Chongqing Municipal Education Commission (KJQN20220045).

## Ethical approval

None.

## Financial disclosure statement

None.

## Trial registration declaration

We solemnly declare that we have not undergone any form of registration or enrollment. Therefore, we are unable to provide any registration name, registration number, or other relevant registration information.

## CRediT authorship contribution statement

**Yuyang Liu:** Writing – review & editing, Writing – original draft, Visualization, Data curation. **Yaheng Tan:** Writing – review & editing, Writing – original draft, Visualization, Data curation. **Jun Wan:** Writing – review & editing, Writing – original draft, Visualization, Supervision, Conceptualization. **Qiwen Chen:** Writing – original draft, Data curation. **Yuxin Zheng:** Visualization, Data curation. **Wenhao Xu:** Visualization, Project administration, Data curation. **Peng Wang:** Visualization, Methodology, Data curation. **Weelic Chong:** Writing – review & editing, Visualization. **Xueying Yu:** Visualization. **Yu Zhang:** Validation, Supervision, Project administration, Methodology, Data curation, Conceptualization.

## Declaration of competing interest

The authors declare that they have no known competing financial interests or personal relationships that could have appeared to influence the work reported in this paper.
